# Impact of Weight Regain on Metabolic Disease Risk: A Review of Human Trials

**DOI:** 10.1155/2014/614519

**Published:** 2014-08-14

**Authors:** Cynthia M. Kroeger, Kristin K. Hoddy, Krista A. Varady

**Affiliations:** Department of Kinesiology and Nutrition, University of Illinois at Chicago, 1919 West Taylor Street, Room 506F, Chicago, IL 60612, USA

## Abstract

Dietary restriction interventions are effective for weight loss and reduction of chronic disease risk. Unfortunately, most people tend to regain much of this lost weight within one year after intervention. While some studies suggest that minor degrees of weight regain have no effect on metabolic disease risk parameters, other studies demonstrate a complete reversal in metabolic benefits. In light of these conflicting findings, it is of interest to determine how complete weight maintenance versus mild weight regain affects key risk parameters. These findings would have important clinical implications, as they could help identify a weight regain threshold that could preserve the metabolic benefits of weight loss. Accordingly, this review examined the impact of no weight regain versus mild regain on various metabolic disease risk parameters, including plasma lipids, blood pressure, glucose, and insulin concentrations, in adult subjects.

## 1. Introduction

It is well known that dietary interventions are effective for weight loss [1, 2]. Most short-term dietary interventions (8–24 weeks) produce body weight decreases of 3–10%, while longer-term protocols (25–52 weeks) produce weight reductions of 10–15% [3–9]. These reductions in body weight are generally accompanied by favorable changes in metabolic disease risk parameters. For instance, weight loss of 5–15% results in fairly consistent decreases in total cholesterol (5–20%) and LDL cholesterol concentrations (5–20%). Beneficial modulations in other key risk parameters, such as systolic blood pressure (5–25%), diastolic blood pressure (5–15%), fasting glucose (5–10%), and insulin (10–40%), are also frequently reported [3–9].

While these weight loss findings are promising, most people tend to regain much of this lost weight within one year after intervention [10–13]. This weight regain is often, but not always, accompanied by a deleterious regression in disease risk towards baseline or beyond [14–16]. More specifically, some studies demonstrate sustained improvements in plasma lipids and glucoregulatory factors with minor weight regain, while others report a complete reversal towards baseline. In light of these conflicting findings, it is of interest to determine how complete weight maintenance versus mild weight regain affects key risk parameters, such as plasma lipids, blood pressure, fasting glucose, and insulin. These findings would have important clinical implications, as they could help identify the weight regain threshold that could preserve the metabolic benefits of weight loss.

Accordingly, this review examined the impact of no weight regain versus mild regain on various metabolic disease risk parameters, including plasma lipids, blood pressure, glucose, and insulin concentrations, in adult subjects.

## 2. Methods

We performed a MEDLINE search in PubMed using various combinations of the following keywords: weight loss, weight loss maintenance, successful weight loss maintenance, weight regain, obese, overweight, cholesterol, glucose, insulin, coronary heart disease, and metabolic syndrome. Inclusion criteria were as follows: (1) randomized control trials and nonrandomized trials, (2) total sample size ≥8 subjects, (3) primary endpoints of body weight and two or more relevant metabolic disease risk parameters, (4) weight loss phase duration ≥4 weeks, (5) weight loss maintenance phase duration ≥4 weeks, (6) male or female subjects, (7) adult subjects ≥18 y, (8) trials reporting preweight loss, postweight loss, and postweight maintenance data, with within-group statistical comparisons made between each time point. Exclusion criteria: (1) cohort and observational studies, (2) trials combining weight loss or weight loss maintenance protocols with supplements or pharmacological substances, (3) trials including subjects with a history of diabetes, coronary heart disease, or cancer, (4) trials including subjects taking lipid- or glucose-lowering medications, and (5) trials reporting continued weight loss during the weight maintenance phase. Twelve studies were found that matched these criteria [17–28].

## 3. Studies Reporting No Weight Regain

We found three studies that showed complete weight maintenance after a period of successful weight loss [17–19]. The effects of no weight regain on plasma lipids, blood pressure, glucose, and insulin are summarized in Table 1.

### 3.1. Plasma Lipids

#### 3.1.1. Total and LDL Cholesterol

Changes in total cholesterol and LDL cholesterol were reported in two studies [17, 19]. While these two studies show consistent decreases in total cholesterol (15-16%) during the weight loss period, changes in this lipid parameter were variable during the weight maintenance period, despite both studies reporting no weight regain. For instance, Claessens et al. [17] demonstrated 14–18% increases in total cholesterol after 12 weeks of a maintenance diet with ad libitum food intake. In contrast, Soenen et al. [19] reported no changes in total cholesterol levels with 17 weeks of diet that provided 100% maintenance energy requirements. One factor that may account for these discrepancies between studies is difference in energy intake. The study by Claessens et al. [17] allowed for ad libitum energy intake and observed increases in total cholesterol. Soenen et al. [19], on the other hand, ensured that their subjects were only consuming energy intake to maintain weight. Thus, the added energy intake in the Claessens study [17], may have contributed to increases in total cholesterol levels.

Changes in LDL cholesterol in the studies by Claessens et al. [17] and Soenen et al. [19] paralleled those of total cholesterol. For example, both studies demonstrated impressive decreases in LDL cholesterol (16–21%) during the weight loss phase, but these reductions were only maintained in the study by Soenen et al. [19]. Though the reason for these differences is still unclear, it is likely that the controlled energy intake by Soenen et al. [19] helped to preserve reductions in LDL cholesterol.

#### 3.1.2. HDL Cholesterol

The impact of weight loss and weight maintenance on HDL cholesterol levels was reported in all three studies in this category [17–19]. During the weight loss phase, HDL cholesterol was unchanged in the studies by Straznicky et al. [18] and Soenen et al. [19] and decreased in the study by Claessens et al. [17]. During the weight maintenance phase, HDL cholesterol remained unchanged in the studies by Straznicky et al. [18] and Soenen et al. [19] and increased in the study by Claessens et al. [17]. One factor that could have impacted these results is exercise. Augmented physical activity is known to increase plasma HDL cholesterol concentrations [29]. Physical activity was not carefully controlled in any of these studies [17–19]. Thus, it is possible that the subjects in the Claessens et al. study [17] increased their physical activity during the weight maintenance arm, which may have contributed to these elevations in HDL cholesterol. Increases in physical activity by these subjects would also explain why fat free mass increased during the weight maintenance phase [17]. Nonetheless, these discrepant findings for HDL cholesterol highlight the need for future trials in this area to carefully monitor exercise, as it can have a major confounding influence on this lipid parameter.

#### 3.1.3. Triglycerides

Consistent decreases in triglyceride concentrations (18–33%) were noted in all three trials during the weight loss period [17–19]. These reductions in triglycerides were sustained in all studies, with the exception of the Claessens et al. study [17], which demonstrated additional reductions (5%). These continued improvements in triglycerides by Claessens et al. [17] may be related to the additional decreases in waist circumference observed during the weight maintenance period. Waist circumference is an indirect marker of visceral fat mass and is positively correlated with circulating levels of triglycerides [30]. Thus, the added reductions in waist circumference may have contributed to these continued improvements in triglyceride concentrations. It should also be noted that the Claessens et al. [17] subjects were consuming an ad libitum-high protein diet (35% kcal as protein) during weight maintenance. Recent findings suggest that high protein diets may be more effective for triglyceride-lowering than regular protein diets (15% kcal as protein) [31, 32]. Thus, macronutrient composition may have also played a role here.

### 3.2. Blood Pressure

Fairly consistent decreases in systolic and diastolic blood pressure (6–9%) were observed during the weight loss period of all three trials [17–19]. These reductions in blood pressure were maintained during the weight maintenance period, with the exception of the Claessens et al. study [17], which demonstrated further decreases. It should be mentioned that the Claessens et al. subjects were consuming a high protein (35% kcal as protein) maintenance diet. Evidence suggests that high protein diets (30–35% kcal as protein) may result in greater blood pressure reductions than regular protein diets (15% kcal as protein) [32–34]. Thus, high protein intake may have played a role in these cardiovascular benefits. Interestingly, the study by Soenen et al. [19] also implemented a high protein diet, but these favorable changes in blood pressure were not observed. Upon further examination, it was shown that the protein intake by Soenen et al. [19] was much lower (22% kcal as protein) than that of Claessens et al. [17] (35% kcal as protein). Thus, it is possible that percent protein intake must exceed 30% for these modulations to occur [32–34].

### 3.3. Glucose

Fasting glucose was unaltered with weight loss and weight maintenance in the studies by Soenen et al. [19] and Straznicky et al. [18]. In the study by Claessens et al. [17], on the other hand, glucose decreased with weight loss and then increased with a high protein weight maintenance diet. Previous research has shown that high protein diets (>30% kcal as protein) lead to increased hepatic glucose output, potentially due to increased gluconeogenesis [35]. Therefore, the high protein content of the maintenance diet may have contributed to this increase in glucose concentrations.

### 3.4. Insulin

Fasting insulin concentrations were measured in two of the three studies in this category. Although the data are very limited, consistent decreases in insulin (6–24%) were observed with weight loss, and these reductions were sustained with no weight regain [17, 19]. While these findings are promising, more studies are undoubtedly needed before solid conclusions can be reached regarding the impact of complete weight maintenance on insulin concentrations.

## 4. Studies Reporting Weight Regain

We retrieved nine studies that demonstrated weight regain after a period of successful weight loss [20–28] (Table 1).

### 4.1. Plasma Lipids

#### 4.1.1. Total and LDL Cholesterol Concentrations

These lipid parameters were measured in three studies that reported mild weight regain (2%) [21–23]. In the study by Lien et al. [22], total and LDL cholesterol concentrations did not change during the weight loss phase, and remained unchanged during the weight maintenance phase. On the other hand, the trials of Wang et al. [21] and Linna et al. [23] demonstrated decreases in total cholesterol (17%–21%) during the weight loss phase. Despite a mere 2% weight regain, total cholesterol rebounded past baseline levels in both studies [21, 23]. The reason for this sharp rebound effect is unclear, but may be related to the extreme weight loss produced by the studies in very short periods of time [21, 23]. For instance, both Wang et al. [21] and Linna et al. [23] observed very pronounced decreases in body weight (12–14%) after only 8 weeks of a 60% calorie restriction regimen. In view of this, it is possible that total cholesterol concentrations did not have a chance to stabilize post-weight loss, in turn allowing lipid concentrations to quickly rebound with minimal weight regain.

Total and LDL cholesterol concentrations were also measured in four studies that reported slightly greater weight regain (4–6%) [24, 25, 27, 28]. Reductions in total cholesterol (10–13%) and LDL cholesterol concentrations (12–15%) were noted during the weight loss phases of these trials [24, 25, 28]. One exception would be the Delbridge et al. study [27], which showed no effect on LDL cholesterol concentrations with 15% weight loss. During the weight maintenance phases of these studies, decreases in total and LDL cholesterol concentrations completely disappeared [24, 25, 28]. Remarkably, this complete reversal in total and LDL cholesterol concentrations occurred even though body weight did not return to baseline in the majority of these studies [24, 25, 28]. These findings suggest that even mild weight regain can have a negative impact on circulating total and LDL cholesterol concentrations, even when some degree of weight loss is maintained.

#### 4.1.2. HDL Cholesterol

HDL cholesterol was assessed in two studies that reported 2% weight regain [22, 23]. Neither study observed changes in HDL cholesterol levels during the weight loss phase [22, 23]. As for the weight maintenance phase, Lien et al. [22] observed no change in HDL cholesterol with a 2% regain. In contrast, Linna et al. [23] demonstrated increases in HDL cholesterol (16%) with the same amount of weight regain (2%). It should be noted that neither of these studies monitored physical activity during weight maintenance. Moreover, the maintenance period was much longer in the Linna et al. study (104 weeks) [23], when compared to Lien et al. (24 weeks) [22]. Thus, it is possible that the Linna et al. subjects increased their activity levels during this long follow-up period, which may have favorably impacted HDL cholesterol levels.

Four studies that reported greater weight regain (4–6%) measured HDL cholesterol levels [24, 25, 27, 28]. Changes in HDL cholesterol during weight loss were highly variable in these trials. Delbridge et al. [27] and Matsuo et al. [28] saw no change in HDL cholesterol with weight loss. In contrast, Márquez-Quiñones et al. [24] and Thomas et al. [25] demonstrated decreases (2–8%) in this lipid parameter. Interestingly, during the period weight maintenance, HDL cholesterol increased in all of these trials [24, 25, 27, 28]. These surprising improvements in HDL cholesterol (5–20%), despite moderate degrees of weight regain, may have occurred due to sustained reductions in waist circumference (i.e., visceral fat mass). Both waist circumference and visceral fat mass (measured directly by MRI) are negatively correlated with HDL cholesterol levels [36]. Thus, it is possible that moderate degrees of weight regain do not have deleterious effects on HDL cholesterol, if net reductions in waist circumference are sustained.

#### 4.1.3. Triglycerides

This lipid parameter was assessed in three of the four studies reporting 2% weight regain [21–23]. Lien et al. [22] reported no change in triglycerides during the weight loss or weight maintenance periods. Wang et al. [21] demonstrated a 27% decrease in triglycerides during the weight loss phase, followed by a 27% increase during the weight maintenance phase. Triglyceride concentrations also decreased by 28% in the study by Linna et al. [23] during the weight loss period and were sustained during the maintenance period, despite a 2% weight regain. It is not clear why triglyceride reductions were maintained in the study by Linna et al. [23] and not in the study by Wang et al. [21]. It was speculated previously that the Linna et al. [23] subjects may have increased their level of physical activity during the long follow-up period. This augmented level of activity may have had a positive impact on triglyceride and HDL cholesterol concentrations [37–39]. This is purely conjecture, however, as physical activity was not monitored, and fat free mass (which tends to increase with activity) was not measured. This once again highlights the need for accurate activity monitoring during dietary intervention trials.

Triglyceride concentrations were assessed in four studies that reported greater weight regain (4–6%) [25, 27, 28]. Fairly consistent reductions in circulating triglycerides (13–47%) were noted with weight loss [25, 27, 28]. During the weight maintenance phase, however, triglycerides rebounded close to baseline or beyond in most trials. This rebound effect occurred even in the studies of Delbridge et al. [27] and Matsuo et al. [28], who still observed a net weight loss at the end of the maintenance period. The only trial that demonstrated sustained reductions in triglycerides, despite a 6% weight regain, was the study by Thomas et al. [25]. It is difficult to determine why lipid improvements were maintained in this trial [25] versus the others [27, 28], since very little information was provided regarding the weight maintenance interventions. One factor that sets the Thomas et al. study [25] apart from the others, however, is the length of the follow-up period. Thomas et al. [25] implemented a rather short follow-up period (20–24 weeks), while the other trials [27, 28] implemented a much longer follow-up period (52–104 weeks). Thus, it can speculated that the short follow-up period by Thomas et al. [25] was not long enough to allow for triglycerides to rebound to baseline levels. It will be of interest for future studies in this area to evaluate the time-course effects of long-term weight maintenance on triglyceride concentrations.

### 4.2. Blood Pressure

This risk parameter was assessed in two studies that reported minimal weight regain [21, 23]. Wang et al. [21] demonstrated decreases in systolic and diastolic blood pressure (8 and 11%, resp.) with weight loss, but these values returned close to baseline with 2% weight regain. On the other hand, Linna et al. [23] observed decreases in diastolic blood pressure (9%) with weight loss, while systolic values remained unaffected. During the weight maintenance phase of the Linna et al. study [23], systolic values rose by 3% and diastolic values returned to baseline with 2% weight regain. Thus, even small amounts of weight regain (2%) may completely reverse the beneficial effects of weight loss on blood pressure. These findings warrant further investigation into the effects of minor weight regain on this vascular parameter.

Systolic and diastolic blood pressures were measured in five trials that reported greater weight regain [24–28]. Blood pressure decreased with weight loss in each of the trials [24–28]. However, with 4–6% weight regain, blood pressure values returned close to baseline in most of the studies. The only exception would be the study by Márquez-Quiñones et al. [24], who demonstrated no change in diastolic blood pressure during the weight loss or weight maintenance phase. It is unclear why some studies demonstrated a complete return to baseline [24, 28], while others showed only a partial return to baseline [25–27]. This effect does not seem to be dependent on trial duration or degree of weight regain. It is possible, however, that background diet composition may have played a role in these variable findings. Evidence suggests that lower salt intake and higher potassium intake during periods of weight maintenance may help sustain blood pressure reductions [40]. Unfortunately, subject dietary intake was not reported during the maintenance phases of any of these trials. As such, the role that diet composition played in these blood pressure changes is not decipherable. This highlights the need for future trials in this field to accurately report food intake throughout the course of the trial.

### 4.3. Glucose

This parameter was assessed in three of the four studies reporting 2% weight regain. Consistent reductions in fasting glucose concentrations (5–10%) were noted with weight loss in the trials by Crujeiras et al. [20], Wang et al. [21], and Linna et al. [23]. In all of these studies [20, 21, 23], glucose concentrations returned close to baseline during the maintenance phase, in response to a 2% weight regain. Increases in waist circumference (2-3%) [20, 23] and fat mass (10%) [23] were also observed during the period of weight maintenance. Evidence suggests that waist circumference and fat mass are directly correlated to fasting glucose concentrations [41, 42]. Thus, it is possible that elevations in body fat and abdominal fat during the maintenance phase may have played a role in the return of glucose values back to baseline.

Only three studies that reported greater weight regain (4–6%) assessed fasting glucose [24, 25, 28]. During the weight loss period, consistent reductions in glucose concentrations were noted for all three studies [24, 25, 28]. During the weight maintenance period, these reductions were sustained in the trials by Matsuo et al. [28] and Thomas et al. [25] but returned to baseline in the trial by Márquez-Quiñones et al. [24]. The reason for these inconsistent findings is unclear and does not appear to be related to either trial duration or degree of weight regain.

### 4.4. Insulin

Insulin concentrations were assessed in two studies that demonstrated 2% weight regain [20, 22]. Fasting insulin decreased with weight loss in both of these studies by 18–31%. During weight maintenance, insulin concentrations returned to baseline in the study by Crujeiras et al. [20] but were sustained in the study by Lien et al. [22]. Interestingly, these inconsistent findings occurred despite 2% weight regain in both studies [20, 22]. It should be noted however that Lien et al. [22] had participants undergo intensive behavioral counseling during the maintenance phase, while Crujeiras et al. [20] did not. The behavioral intervention implemented by Lien et al. [22] emphasized the dietary approaches to stop hypertension (DASH) dietary pattern [33]. A recent meta-analysis suggests that the DASH diet reduces fasting insulin levels when prescribed for more than 16 weeks [43]. Therefore it is possible that insulin reductions were sustained in part because of the background diet imposed by Lien et al. [22]. Additionally, Lien et al. [22] demonstrated sustained decreases in visceral fat mass, while Crujeiras et al. [20] reported increases. Visceral fat mass is a key determinant in fasting insulin levels [30]. This factor may have also played a role in the sustained reductions in insulin by Lien et al. [22].

Insulin concentrations were only measured in two studies reporting greater weight regain [24, 25]. Both of these studies demonstrated impressive reductions in insulin (25–30%) with weight loss and a partial return to baseline with weight regain [24, 25]. In examining the data more closely, it would appear as though the degree of weight regain played a role in the size of this rebound effect. For instance, in the trial by Márquez-Quiñones et al. [24], subjects gained back only half of their baseline body weight, and insulin only rebounded slightly. On the other hand, in the study by Thomas et al. [25], subjects gained back almost all of their body weight, and insulin levels rebounded almost completely. Thus, small amounts of weight regain may allow for a partial maintenance of insulin-lowering, while larger amounts of weight regain may cause the insulin-lowering effect to disappear.

## 5. Summary of Findings

*Studies Reporting No Weight Regain*. Whether or not reductions in total and LDL cholesterol are maintained with no weight regain cannot be elucidated, as the evidence is sparse and equivocal [17, 19]. As for HDL cholesterol, neither weight loss nor complete weight maintenance appear to have any effect on this lipid parameter [17–19]. Triglyceride concentrations, on the other hand, tend to decrease with weight loss, and these reductions are sustained with no weight regain [17–19]. The same holds true for blood pressure and insulin levels. Although the data are limited, it would appear as though reductions in blood pressure and insulin seen with weight loss are maintained when an individual regains no weight [17–19]. Fasting glucose levels, in contrast, do not appear to decrease with weight loss and are unaltered during periods of complete weight maintenance [17–19].

*Studies Reporting Weight Regain*. The majority of trials in this category demonstrate that total cholesterol, LDL cholesterol, and triglyceride concentrations return to baseline when 2–6% of weight is regained [21–25, 27, 28]. As for HDL cholesterol, surprising improvements in this lipid parameter were noted with sustained decreases in visceral fat (even with overall weight regain) [24, 25, 27, 28]. With regards to blood pressure, glucose, and insulin, most of the benefits of weight loss seem to disappear with mild weight regain [23–28]. Remarkably, these rebound effects for each parameter occurred even though body weight did not return to baseline in the majority of studies.

## 6. Limitations

This review has several limitations. Firstly, the interventions, trial durations, and populations between studies are quite heterogeneous. This heterogeneity makes it difficult to draw clear conclusions from the data as a whole and needs to be taken into consideration when interpreting the findings. Secondly, the number of studies that met our inclusion and exclusion criteria was very limited. Thus, it is not possible to make clear-cut clinical recommendations, as the data are far too sparse. Thirdly, the studies included here did not report whether or not measurements were taken during periods of weight stability. Therefore, we cannot eliminate the potential confounding effects that negative or positive energy balance had on the reported parameters. Fourthly, the studies in the “no weight regain” category had very short weight maintenance periods (12–17 weeks) when compared to the “weight regain” category (24–104 weeks). These short intervention periods may not have been long enough to allow for these parameters to stabilize/rebound fully and should be considered when drawing conclusions from these data.

## 7. Conclusion

Taken together, these preliminary findings suggest that improvements in metabolic risk factors with weight loss may only be sustained with no weight regain. Although the data are very limited, it would appear as though even mild degrees of weight regain (i.e., 2–6%) can cause plasma lipids, blood pressure, fasting glucose, and insulin concentrations to revert back to baseline or beyond. Further investigation is undoubtedly warranted to identify the precise degree of weight maintenance that would be required to preserve the metabolic benefits of weight loss.

## Figures and Tables

**Table 1:** Effect of weight loss and weight maintenance interventions on body weight and metabolic disease risk.

Reference	Subjects	Interventions	Length	Weight loss %	Weight regain %	Body composition				Lipids				Blood pressure		Glucose	Insulin
			Weeks			FM	FFM	VFM	WC	Total	LDL	HDL	TAG	Systolic	Diastolic		
Studies reporting no weight regain∗																	
Claessens et al., 2009 [17]	*n* = 14 MF	**Wt loss: **50% CR	5	↓ 9%	—	↓ 18%	↓ 3%	—	↓ 9%	↓ 16%	↓ 21%	↓ 9%	↓ 19%	↓ 6%	↓ 6%	↓ 7%	↓ 24%
	45 ± 2 y	**Wt maint: **Ad libitum-HP	12	—	*∅*	↓ 6%	↑ 2%		↓ 2%	↑ 14%	↑ 14%	↑ 20%	↓ 5%	↓ 4%	↓ 3%	↑ 8%	*∅*
	Obese	**Wt maint:** Ad libitum	12	—	*∅*	*∅*	↑ 2%		*∅*	↑ 18%	↑ 17%	↑ 17%	*∅*	*∅*	*∅*	*∅*	*∅*
Straznicky et al., 2011 [18]	n = 8 MF	**Wt loss: **25% CR	12	↓ 7%	—	—	—	—	↓ 7%	—	—	*∅*	↓ 33%	↓ 8%	*∅*	*∅*	—
	54 ± 2 y	**Wt maint: **100% needs	16	—	*∅*				*∅*			*∅*	*∅*	*∅*	*∅*	*∅*	
	Obese																
Soenen et al., 2013 [19]	n = 36 MF	**Wt loss: **33% CR	6	↓ 7%	—	↓ 13%	*∅*	—	—	↓ 15%	↓ 16%	*∅*	↓ 18%	↓ 9%	↓ 6%	*∅*	↓ 6%
	44 ± 4 y	**Wt maint: **100% needs-HP	17	—	*∅*	↓ 7%	↑ 1%			*∅*	*∅*	*∅*	*∅*	*∅*	*∅*	*∅*	*∅*
	Obese	**Wt maint: **100% needs	17	—	*∅*	↓ 6%	↑ 1%			*∅*	*∅*	*∅*	*∅*	*∅*	*∅*	*∅*	*∅*

Studies reporting weight regain∗																	
Crujeiras et al., 2014 [20]	n = 61 MF	**Wt loss: **50% CR	8	↓ 6%	—	—	—	—	↓ 5%	—	—	—	—	—	—	↓ 5%	↓ 31%
	42 ± 1 y	**Wt maint: **Not specified	24	—	↑ 2%				↑ 2%							↑ 8%	↑ 33%
	Obese																
Wang et al., 2012 [21]	n = 125 M	**Wt loss: **60% CR	8	↓ 12%	—	↓ 25%	—	—	—	↓ 17%	—	—	↓ 27%	↓ 8%	↓ 11%	↓ 6%	—
	43 ± 6 y	**Wt maint: ** Not specified	24	—	↑ 2%	*∅*				↑ 23%			↑ 27%	↑ 6%	↑ 5%	↑ 2%	
	Obese																
Lien et al., 2009 [22]	n = 27 MF	**Wt loss: **30% CR	24	↓ 6%	—	↓ 11%	↓ 2%	↓ 22%	—	*∅*	*∅*	*∅*	*∅*	—	—	—	↓ 18%
	51 y	**Wt maint: **Behavioral	24	—	↑ 2%	↑ 7%	*∅*	*∅*		*∅*	*∅*	*∅*	*∅*				*∅*
	Obese	therapy + low salt diet															
Linna et al., 2007 [23]	n = 68 M	**Wt loss: **60% CR	8	↓ 14%	—	↓ 30%	—	—	↓ 14%	↓ 21%	—	*∅*	↓ 28%	*∅*	↓ 9%	↓ 10%	—
	43 ± 5 y	**Wt maint: ** Not specified	104	—	↑ 2%	↑ 10%			↑ 3%	↑ 22%		↑ 16%	*∅*	↑ 3%	↑ 8%	↑ 6%	
	Obese																
Sumithran et al., 2011 [26]	*n* = 34 MF	**Wt loss: **50% CR	10	↓ 14%	—	—	—	—	↓ 11%	—	—	—	—	↓ 10%	↓ 11%	—	—
	56 ± 11 y	**Wt maint: **Not specified	52	—	↑ 4%				↑ 4%					↑ 2%	↑ 6%		
	Obese																
Delbridge et al., 2009 [27]	n = 141 MF	**Wt loss: **50% CR	12	↓ 15%	—	—	—	—	↓ 8%	↓ 12%	*∅*	*∅*	↓ 45%	↓ 10%	↓ 10%	—	—
	44 ± 1 y	**Wt maint: **Ad libitum-HP	52	—	↑ 4%				*∅*	↑ 9%	*∅*	↑ 14%	↑ 19%	↑ 4%	↑ 5%		
	Obese																
Matsuo et al., 2010 [28]	*n* = 54 F	**Wt loss: **30% CR	14	↓ 13%	—	↓ 29%	↓ 3%	↓ 29%	—	↓ 13%	↓ 14%	*∅*	↓ 47%	↓ 10%	↓ 12%	↓ 8%	—
	56 ± 5 y	**Wt maint: **Not specified	104	—	↑ 4%	↑ 13%	*∅*	*∅*		↑ 14%	↑ 14%	↑ 9%	↑ 44%	↑ 10%	↑ 9%	*∅*	
	Obese																
Márquez-Quiñones et al., 2010 [24]	*n* = 38 MF	**Wt loss: **25% CR	8	↓ 10%	—	—	—	—	↓ 8%	↓ 10%	↓ 15%	↓ 8%	*∅*	↓ 7%	*∅*	↓ 6%	↓ 30%
	42 ± 1 y	**Wt maint: **Not specified	24	—	↑ 5%				↑ 4%	↑ 8%	↑ 12%	↑ 20%	*∅*	↑ 6%	*∅*	↑ 4%	↑ 8%
	Obese																
Thomas et al., 2010 [25]	*n* = 67 MF	**Wt loss: **25% CR	20—24	↓ 7%	—	—	—	↓ 26%	↓ 8%	↓ 11%	↓ 12%	↓ 2%	↓ 13%	↓ 6%	↓ 5%	↓ 3%	↓25%
	40 ± 1 y	**Wt maint: **Not specified	20—24	—	↑ 6%			↑ 23%	↑ 4%	↑ 12%	↑ 13%	↑ 5%	*∅*	↑ 3%	*∅*	*∅*	↑ 20%
	Obese																

∗studies ordered by ascending degree of weight regain.

*∅*: no significant change, CR: calorie restriction, F: female, FFM: fat free mass, FM: fat mass, HP: high protein diet, M: male, TAG: triglycerides, WC: waist circumference, VFM: visceral fat mass by MRI or CT.
